# Current approaches for the exploration of antimicrobial activities of nanoparticles

**DOI:** 10.1080/14686996.2021.1978801

**Published:** 2021-10-15

**Authors:** Nur Ameera Rosli, Yeit Haan Teow, Ebrahim Mahmoudi

**Affiliations:** a Department of Chemical and Process Engineering, Faculty of Engineering and Built Environment, Universiti Kebangsaan Malaysia, Bangi, Malaysia; b Research Centre for Sustainable Process Technology (Cespro), Faculty of Engineering and Built Environment, Universiti Kebangsaan Malaysia, Bangi, Malaysia

**Keywords:** Nanoparticles, antimicrobial, antiviral, mechanism, applications, 30 Bio-inspired and biomedical materials, 200 Applications, 306 Thin film / Coatings, 300 Processing / Synthesis and Recycling

## Abstract

Infectious diseases of bacterial and viral origins contribute to substantial mortality worldwide. Collaborative efforts have been underway between academia and the industry to develop technologies for a more effective treatment for such diseases. Due to their utility in various industrial applications, nanoparticles (NPs) offer promising potential as antimicrobial agents against bacterial and viral infections. NPs have been established to possess potent antimicrobial activities against various types of pathogens due to their unique characteristics and cell-damaging ability through several mechanisms. The recently accepted antimicrobial mechanisms possessed by NPs include metal ion release, oxidative stress induction, and non-oxidative mechanisms. Another merit of NPs lies in the low likelihood of the development of microbial tolerance towards NPs, given the multiple simultaneous mechanisms of action against the pathogens targeting numerous gene mutations in these pathogens. Moreover, NPs provide a fascinating opportunity to curb microbial growth before infections: this outstanding feature has led to their utilization as active antimicrobial agents in different industrial applications, e.g. the coating of medical devices, incorporation in food packaging, promoting wound healing and encapsulation with other potential materials for wastewater treatment. This review discusses the progress and achievements in the antimicrobial applications of NPs, factors contributing to their actions, mechanisms underlying their efficiency, and risks of their applications, including the antimicrobial action of metal nanoclusters (NCs). The review concludes with a discussion of the restrictions on present studies and future prospects of nanotechnology-based NPs development.

## Graphical abstract

**Figure:**
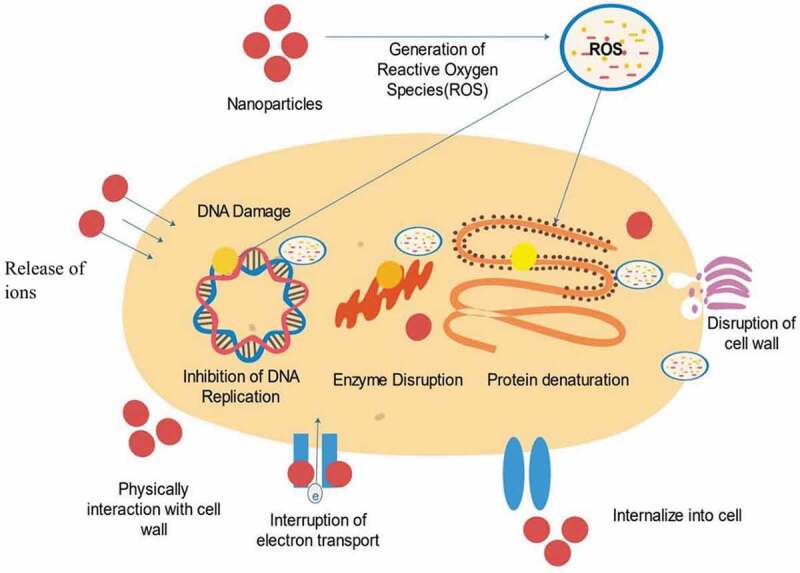

## 1. Introduction

Infectious diseases of bacterial and viral origin present a major medical concern across the globe, given the substantial mortality and morbidity associated with them. In developing countries, approximately 50% of the community is infected with bacterial infections, and beyond 3 million deaths are recorded annually [1]. Bacteria can be found easily everywhere and their attachment to surfaces is usually followed by the formation of biofilms. Such biofilms confer resistance against antibiotics, due to which it is clinically challenging to cure the infections [2]. Moreover, the biofilms enhance corrosion on metallic surfaces and provoke mechanical blockage in fluid systems [3,4]. The presence of bacteria promotes colonization in operation theatres and on medical devices, leading to nosocomial infections. Viruses, on the other hand, tend to replicate in their cellular hosts, wherein their evolving genomes affect metabolic pathways in the host cells, leading to clinical infections [5]. Hence, it is crucial to develop technologies to combat such pathogenic infections: in this regard, antimicrobial agents play an important role.

Nanoparticles (NPs) have been hailed as a novel alternative to treat infections. In terms of structure, NPs are materials with a three-dimensional basic unit that falls within the range between 1 and 100 nm [6]. The physicochemical properties of NPs underlying their antimicrobial activities include their size, charge, surface morphology, crystal structure, and zeta potential [7]. A small dimension of size is the main advantage of NPs to achieve excellent antimicrobial actions and to effectively combat intracellular bacteria, as it facilitates the penetration of NPs through bacterial cell walls into the bacteria [2]. When NPs attach and bind electrostatically to bacterial cell walls, membrane damage can occur to the bacteria, leading to membrane potential alteration and depolarization. This causes the bacterial cells to lose their integrity, resulting in impaired respiration, imbalance of ions within the bacteria, interruption of energy transduction, and eventually cellular lysis [8].

Copious research has been conducted on the physicochemical properties of NPs. In terms of size, a study on *Methylobacterium* spp revealed that smaller silver (Ag) NPs (10 nm) displayed higher cytotoxicity than larger NPs (100 nm) at the same concentration (1.0 mg/mL) [9]. Additionally, the smaller NPs release silver ions (Ag^+^) due to their larger surface areas and hence exhibit stronger antimicrobial activity, compared to their larger counterparts [9]. In terms of surface morphology, smooth surfaces have been found to enable less bacterial adhesion rough ones, which increase the potential of NPs to be in contact with bacterial cells due to more regions where the bacteria can bind favorably [10,11]. A perfectly smooth surface would be less likely to be inhabited by microorganisms than a rough surface, where a greater surface area is available on which the microorganism will produce more adhesive force per surface area [12].

It has been established that bacterial adhesion depends on the surface charges of NPs and their zeta potential. Negatively charged and neutral Ag NPs exhibited intermediate antimicrobial activities, whereas positively charged ones exhibited the greatest antimicrobial activities upon all microorganisms tested [13,14]. This observation can be attributed to the presence of negatively charged peptidoglycans in bacterial cell walls, towards which the positively charged NPs are attracted [15,16]. The zeta potential of NPs reflects the stability of NP-based suspensions due to its relation to particulate aggregation: a large negative or large positive zeta potential for an NP-based suspension implies substantial stability, given the negligibly small aggregation. Such stability of NPs will eventually enhance their antimicrobial activities. In terms of other factors, environmental factors such as the bacterial strain used and the exposure time towards NPs also govern the antimicrobial activities of NPs [7]. The longer the exposure time of NPs onto a living microorganism, the greater the antimicrobial activity. Collectively, these unique properties facilitate the antimicrobial mechanisms of NPs in combating bacterial infections, thus underlining their potential as efficacious antimicrobial agents.

The application of these NPs as antimicrobial agents in medicine has attracted scholarly attention in developing novel pharmaceutical nanotechnology [17]. NPs demonstrate broad-spectrum antimicrobial activity against Gram-positive and Gram-negative bacteria, depending on the type of NPs. Additionally, NPs can offer an alternative way to produce collective disinfectants by integrating NPs into industrial and clinical devices. To date, a PubMed search of ‘antimicrobial nanoparticles’ from 5 years of publication dates has resulted in 14,267 articles, including 1,191 review articles focusing on the antimicrobial activities of NPs. Table 1 outlines the efficacy of several NPs at different concentrations based on their zones of inhibition obtained from the disk diffusion method. The zone of inhibition is the circular area around the spot of the NPs in which the bacterial colonies do not grow and indicates the antimicrobial activity of NPs. Some of the most well-known NPs are silver, gold, and platinum. However, the antibacterial mechanisms of NPs have not been thoroughly explained due to a lack of unified standards to compare antimicrobial activities, since different parameters have been used in most existing research for the type of bacterial strain used, action times, and NPs characteristics.

**Table 1.** Zone of inhibition of several NPs at different concentrations of various species of bacteria

Antimicrobial NPs	Concentration of NPs (mg/mL)	Type of bacteria	Target bacteria	Zone of inhibition (mm)	References
Silver (Ag)	0.05	Gram-positive	*Enterococcus faecium* *(E. faecium)*	3.00	[27]
	0.50		*Staphylococcus aureus* *(S. aureus)*	14.60	[175]
	0.62		*Listeria monocytogenes* *(L. monocytogenes)*	9.16	[176]
	10.00	Gram-negative	*Yersinia ruckeri* *(Y. ruckeri)*	16.00	
	0.50		*Escherichia coli* *(E. coli)*	23.00	[175]
Gold (Au)	0.05	Gram-positive	*E. faecium*	1.00	[27]
	0.01		*S. aureus*	22.00	[177]
		Gram-negative	*E. coli*	17.00	
Platinum (Pt)	1.00	Gram-positive	*Bacillus substilis (B. subtilis)*	18.00	[178]
		Gram-negative	*Pseudomonas aeruginosa* *(P. aeruginosa)*	15.00	
Copper (Cu)	0.05	Gram-positive	*S. aureus*	10.00	[80]
		Gram-negative	*P. aeruginosa*	16.00	
			*E. coli*	24.00	
			*Klebsiella pneumonia* *(K. pneumoniae)*	27.00	
			*Shigella flexneri* *(S. flexneri)*	30.00	
Magnesium Oxide (MgO)	1.00	Gram-positive	*S. aureus*	27.00	[179]
		Gram-negative	*P. aeruginosa*	24.00	
Zinc Oxide (ZnO)	0.05	Gram-positive	*Methicillin-resistant Staphylococcus aureus* (MRSA)	17.00	[180]
	0.04	Gram-negative	*P. aeruginosa*	35.50	[181]
	1.00		*E. coli*	14.00	[182]
	1.40		*K. pneumoniae*	25.00	[183]
Titanium dioxide (TiO_2_)	0.05	Gram-positive	MRSA	14.00	[180]
	1.40	Gram-negative	*K. pneumoniae*	20.00	[183]
Palladium (Pd)	1.00	Gram-positive	*E. faecium*	6.50	[27]
		Gram-negative	*Acinetobacter baumannii* *(A. baumannii)*	8.20	
			*K. pneumoniae*	10.50	

This review focuses on the antimicrobial and antiviral activities of NPs and their underlying mechanisms in order to derive insights into the development of more practical materials in the future. This review also discusses the antimicrobial properties of metal nanoclusters (NCs).

## 2. Antimicrobial mechanism of nanoparticles

The increasing use of NPs in various industrial fields has paved the way for the investigation of their antimicrobial mechanism. Generally, the antimicrobial activation of NPs is characterized by one of the three models: metal ion release, oxidative stress induction, and non-oxidative mechanism, where most of the mechanisms involve the overproduction of reactive oxygen species (ROS) [7]. Existing recent studies state that the most common processes underlying the antimicrobial actions of NPs include the induction of disruption of cell walls and cell membranes, generation of ROS that leads to oxidative stress, disruption of energy transduction, enzyme inhibition, photocatalysis, and interference in DNA and RNA [18].

### 2.1 Disruption of cell walls and cell membranes

**Figure 1.**
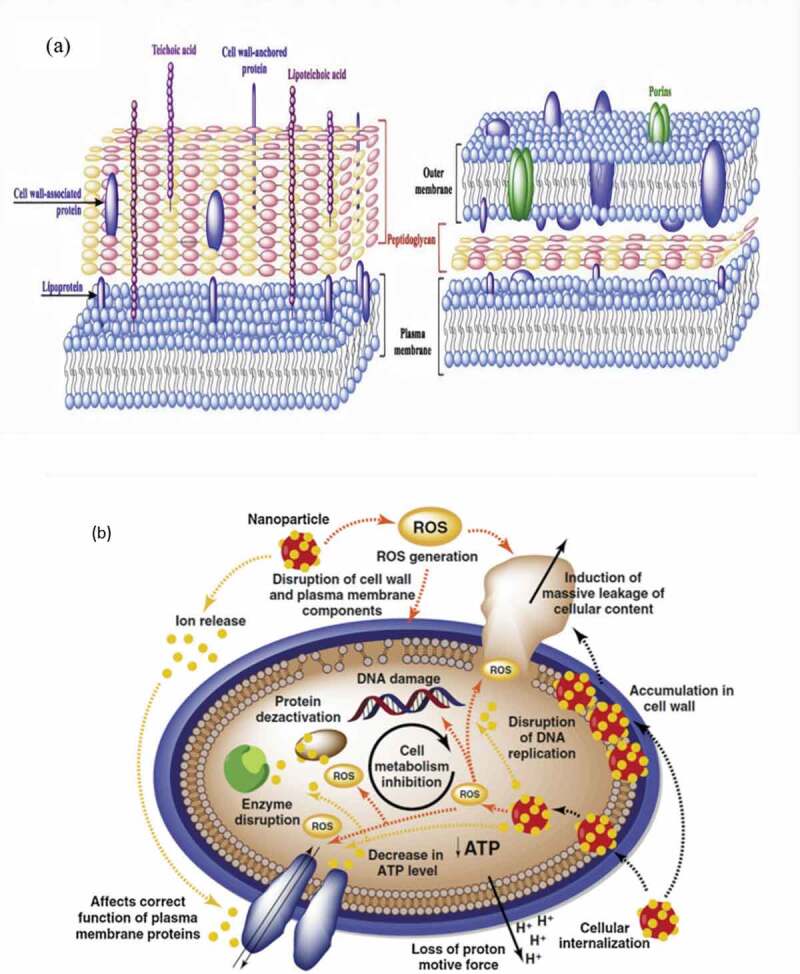
(a) Structural comparison of the cell wall between Gram-positive and Gram-negative bacteria [185] and (b) various mechanisms underlying the antimicrobial actions of nanoparticles [186], copyright 2019 and copyright 2016, Elsevier

The first line of defense of bacteria is represented by their cell walls and cell membranes. Both components hold important roles as a defensive barrier by helping the bacteria to maintain their shape and by providing support and protection from the external environment [19]. Cell lysis is known to be the initial mechanism underlying the antimicrobial action of NPs, which begins by targeting cell walls and damaging cell membranes. The adsorption pathway for NPs is governed by the components of the bacterial cell membrane. The antimicrobial actions of NPs are greater against Gram-positive bacteria than against Gram-negative ones: the cell wall of Gram-negative bacteria comprises lipoproteins, lipopolysaccharides, and phospholipids, which permit the penetration of only macromolecules by forming a penetration barrier between the cell membrane and its surroundings [6,7]. Conversely, the cell wall of Gram-positive bacteria comprises a thick layer of peptidoglycan and teichoic acid with numerous pores that allow easy penetration of foreign molecules, which then leads to membrane disruption, loss of cytoplasmic constituents, and ultimately apoptosis (Figure 1) [20]. Such observations have been reported in the literature. In one study, the number of Au NPs attached to *B. subtilis* was found to be higher (10^3^ NPs) than that to *Stenotrophomonas maltophilia* (*S. maltophilia*) (60 NPs), owing to the different widths of the cell walls of both bacteria [21]. In another, a 10-min exposure of 50 μg/mL Ag NPs to laser light radiation caused the populations of *S. aureus* (Gram-positive) and *E. coli* (Gram-negative) to reduce to 28% and 14%, respectively [22]. Ag NPs have also been reported to break down lipopolysaccharides in cell walls, causing their aggregation in the cell membrane that in turn would form abundant small pits that altered membrane permeability [23,24].

The degree of disruption of the cell walls and cell membranes is related to the size and charge of NPs. For example, the smaller particle size of Ag NPs led to smaller values of the minimum inhibitory concentration (MIC) and minimum bactericidal concentration (MBC), suggesting a superior antimicrobial activity [25]. Moreover, since peptidoglycan (more abundantly present in Gram-positive bacteria) is negatively charged, the positively charged NPs will be attracted to Gram-positive bacteria to a greater extent than to Gram-negative ones [15]. More peptidoglycan indicated that Gram-positive bacteria have an abundance of negative charge compared to Gram-negative bacteria. The disruption of the cell walls and cell membranes can also be attributed to the release of harmful ions by NPs that aid in their antimicrobial activities. Ions released by NPs upon contact with the cell wall will inactivate enzymes and cellular proteins by reacting with their thiol groups, interrupting the metabolic actions, and causing the formation of ROS [23]. Different ions exhibit different properties. Both Au^+^ and Au^3+^ released by Au NPs exerted potent antimicrobial activities against four tested bacteria: one non-pathogenic *E. coli* and three multidrug-resistant bacteria *Salmonella typhimurium* (*S. typhimurium), E. coli*, and MRSA [26]. Zn^2+^ released by ZnO NPs also displayed similar antimicrobial activities; however, low levels of solubilized Zn^2+^ ions can act as a source of nutrients for bacterial growth and can induce tolerance in various microorganisms [23]. Ag NPs release Ag^+^ that is said to be a key contributor to their cytotoxicity as Ag^+^ is known to cause bacteria cell walls to rupture, hinder cellular respiration, prevent DNA replication, and eventually induce DNA damage [23,27,28]. However, opinion is divided on the toxicity of NPs: some evidence has attributed the toxicity of Ag NPs to the Ag^+^ released from Ag NPs, whereas other evidence has attributed it to the NPs themselves [28]. Smaller Ag NPs have also been reported to be associated with a higher rate of intracellular influx of Ag^+^ due to their greater surface-area-to-volume ratio: the liberated Ag^+^ then comes in contact with respiratory chain proteins on the cell membrane, inducing ROS, thus leading to cellular oxidative stress on the cell [29]. Nam et al. have suggested that NPs needed to be in their ionic form to exhibit their antibacterial activities [30]. Hence, the expanded contact region of the NPs will facilitate their generation of more ions to interact with the bacteria and damage them via multiple pathways.

**Figure 2.**
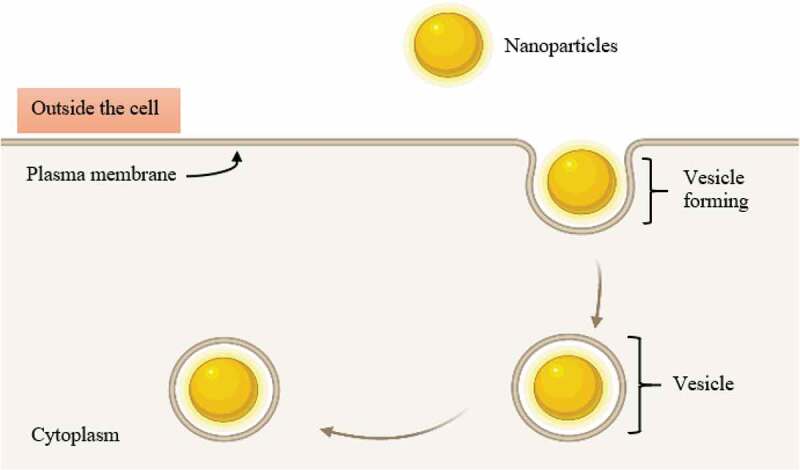
The process of engulfing of NPs by the cell membrane

**Figure 3.**
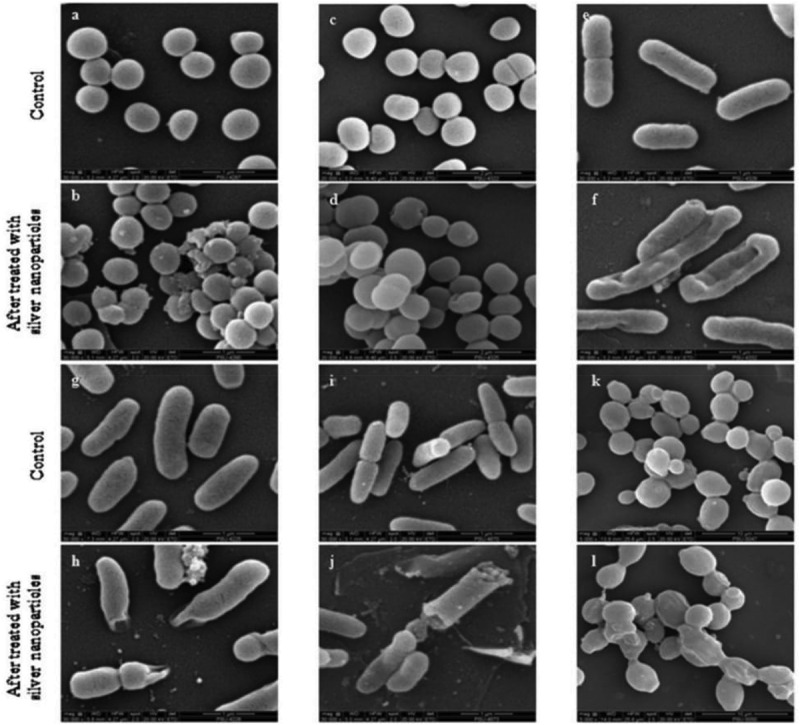
(a) Antimicrobial activity of Ag NPs on *S. aureus* ATCC 25923 viewed through a scanning electron microscope (c), *A. baumannii* ATCC 19606, (e) *E. coli* O157:H7, (g) *K. pneumoniae* ATCC 700603, (i) *P. aeruginosa* ATCC 10145, (k) *C. albicans* ATCC 90028 after exposure for 4 hours to 2 × MIC Ag NPs. The control (a, c, e, g, and i) without treated with Ag NPs, revealing a smooth surface. Cultures (b, d, f, h, j, and l), showing roughed surface with fracture after treated with Ag NPs [34] copyright 2019, Elsevier

After targeting the bacterial cell wall via endocytosis, NPs will further interrupt the bacterial cell membrane. At the beginning of the interruption, NPs can attach themselves to the membrane interface through adhesion and through being partly engulfed by the lipid bilayer [31]. After the engulfing process is complete, NPs will detach themselves from the inner surface of the cell membrane through fission and leave a transient membrane pore (Figure 2) [32]. In this regard, the antimicrobial effects of NPs such Ag- and Au-based NPs have been investigated. For *Ralstonia solanacearum* (*R. solanacearum*), its cell membrane has been reported to become uneven with the presence of distortion in the cell structure after incubation for 3 hours with pure Ag NPs (19.5 mg/L) at 30°C, which ultimately led to microbial death. For *S. aureus*, its cell walls have been reported to have been damaged (Figure 3) due to lysis secondary to a 24-h exposure to Ag NPs [33]. Moreover, another study revealed that treating several bacteria cultures (e.g. *S. aureus, A. baumannii, E. coli, K. pneumoniae, P. aeruginosa*, and *Candida albicans* (*C. albicans*)) with 2 × MIC Ag NPs for 4 hours resulted in a rougher surface to the bacteria and eventually, fractured compared to the untreated cultures (Figure 3) [34]. Au NPs also demonstrated antimicrobial actions: when tested on *S. typhimurium*, Au NPs contributed to membrane disruption and thereby necrotic cell death [35].

Besides, the disruption of bacterial membranes hinders the formation of biofilms, one of the resistance mechanisms contributing to bacterial survival in the presence of antibiotics [36]. A biofilm is defined as a colony of microorganisms that attach to the surfaces and grow within a matrix of extracellular polymeric substances (EPS) produced by them [37,38]. Such bacterial biofilms can cause nosocomial infections and are usually pathogenic [39,40]. It has been reported that treatments for infections associated with biofilms are challenging due to the presence of an EPS matrix surrounding the bacterial cells, which makes them tolerant against antibiotics [41]. In this regard, it has been copiously reported in the literature that NPs can inhibit the formation of biofilms. In one study, the 14-day incubation of Cu NPs with *K. pneumonia* hindered the formation of biofilms, as evidenced by the absence of bacteria or biofilms on the surface of Cu NPs through scanning electron microscopy [42]. In another study, cinnamaldehyde-immobilized Au NPs with a concentration of 125 µg/ml demonstrated biofilm inhibition of approximately 80% against Gram-positive bacteria (e.g. methicillin-sensitive *S. aureus* (MSSA) and MRSA) and Gram-negative bacteria (e.g. *E. coli* and *P. aeruginosa*) [43]. Ag NPs have also been demonstrated to reduce the formation of curli (a type of proteinaceous extracellular surface-associated fibres) by *E. coli* and in turn biofilm formation [44]. Under the Congo red (CR) assay, the *E. coli* changed from a dry, red, and rough morphology to a smooth, pale, and wrinkled morphology after treatment with Ag NPs which indicates that Ag NPs have successfully reduced curli fiber and leads to inhibition of biofilm formation. Furthermore, a combination of silver-graphene (Ag/GO) has proven effective for controlling biofilm formation as field emission scanning electron microscope (FESEM) revealed that 0.8 wt% of Ag/GO nanohybrids inhibited colony formation on surfaces of polyamide 6,6 membranes [45]. Additionally, MgO NPs demonstrated excellent biofilm inhibition: for *E. coli*, membrane damage was observed at concentrations exceeding 0.5 mg/mL for MgO NPs, whereas for *Staphylococcus epidermidis* (*S. epidermidis*), it was observed at 1.6 mg/mL [46]. Biofilm inhibition might be caused by the inhibitory effect on the expression of genes governing both biofilm formation and bacterial motility [41].

### 2.2 Disruption of mitochondrial electron transport chain

Another antimicrobial mechanism of NPs concerns the disruption of the mitochondrial electron transport chain (ETC). The high affinity of Ag NPs and Ag^+^ for thiol groups in cysteine residues has been reported to interrupt mitochondrial membrane proteins, membrane permeability, and mitochondrial functions [47]. ETCs are a series of protein complexes in the mitochondrial inner membrane that couple redox reactions transferring electrons from electron donors to electron receptors through an electrochemical gradient, thereby generating adenosine triphosphates (ATPs) which are essential for cellular respiration [48]. The four complexes in the ETC are complexes I (nicotinamide adenine dinucleotide (NADH) dehydrogenase), II (succinate dehydrogenase), III (cytochrome c oxidoreductase), and IV (cytochrome oxidase) [48]. NPs can accumulate in the mitochondria, resulting in mitochondrial membrane depolarization and blockage of ETC following exposure to NPs through activation of nicotinamide adenine dinucleotide phosphate (NADPH)-related enzyme [49,50]. The blockage of the ETC will further increase the cellular level of ROS via electron transfer [50]. In one study, Cu NPs have been demonstrated to have the potential to block the functions of complexes I and III on the mitochondrial ETC causing the over-generation of ROS and oxidative stress upon cell membranes [48]. In another study, TiO_2_ NPs have been demonstrated to disrupt the ETC as evidenced by the down-regulation of cardiolipin (a phospholipid in the mitochondrial inner membrane responsible for maintaining the functions of those ETC complexes) [51].

### 2.3 Overproduction of reactive oxygen species

Given the central role of mitochondria in regulating the intracellular level of ROS, the disruption of ETC leads to excessive mitochondrial production of ROS [52,53]. Originally known as deleterious metabolic by-products, ROS has now been established to assist multiple functions as intracellular regulators of signaling pathways including maintaining a healthy redox process within cells [54,55]. Controlled ROS production has also been reported to manage processes including initiation of defenses towards pathogens, programmed cell lysis, and generation of energy via the mitochondrial ETC [56]. Table 2 outlines biologically significant ROS elements.

**Table 2.** ROS elements [50,184]

Types	ROS Elements
Free radicals	Singlet oxygen (^1^O_2_), superoxide (O_2_^ׄ•–^), carbonate (CO_3_^•–^), peroxyl (RO_2_^•^), hydroxyl (HO^•^), hydroperoxyl (HO_2_^•^), alkoxyl (RO^•^), and carbon dioxide radical (CO_2_^•–^)
Non-radicals	Hydrogen peroxide (H_2_O_2_), hypobromous acid (HOBr), hypochlorous acid (HOCl), ozone (O_3_), organic peroxides (ROOH), peroxynitrite (ONOO^–^), peroxynitrate (O_2_NOO^–^), peroxynitrous acid (ONOOH), peroxomonocarbonate (HOOCO_2_^–^), nitric oxide (NO), and hypochlorite (OCl^–^)

Given their substantial reactivity, excessive production of ROS can be deleterious for essential biomolecules [57]. For example, for lipids, ROS disables polyunsaturated fatty acids, which will cause structural damage; whereas, for proteins, they cause enzyme inhibition by initiating the oxidation of cofactors and amino acids [23]. Oxidative stress caused by ROS has been reported to be a major contributor to the changes of cell membrane permeability: Ansari et al. have suggested that the interaction of aluminium oxide (Al_2_O_3_) NPs with cells caused membranes lose the integrity, most probably caused by intracellular oxidative stress [7,58]. ROS-induced oxidative stress is an important antimicrobial action of NPs. For instance, MgO NPs can produce O_2_ׄ^•–^, Cu NPs can produce O_2_ׄ^•–, 1^O_2_, HO^•^ and H_2_O_2_, whereas ZnO NPs can produce OH and H_2_O_2_, though not O_2_^ׄ• –^ [7]. H_2_O_2_ and O_2_^ׄ• –^ result in acute stress reactions on the cell membranes. Such reactions can be prevented with the aid of catalase and superoxide enzymes that act as endogenous antioxidants, though ^1^O_2_ and HO^•^ can cause acute microbial death [7]. All forms of iron (Fe^0^, Fe^2+^, Fe^3+^) employed in NPs have the potential to generate ROS in aqueous solutions in the course of Fenton, Haber–Weiss, and heterogeneous redox reactions [59]. Fenton-type reactions involve the removal of one electron from molecular oxygen (O_2_), resulting in the formation of O_2_ׄ^• –^ which generates other ROS species including HO^•^ [23,60]. Meanwhile, Haber–Weiss type reactions involve the interaction between H_2_O_2_ and oxidized metal oxides, resulting in the formation of HO^•^ [23,60,61]. The Fenton reaction (1) of the Haber–Weiss reaction (2) of Fe^2+^ and Fe^3+^ are shown below [57]:

(1) $$H_{2}O_{2}+\;Fe^{2+}\to HO^{∙}+\;HO^{-}+\;Fe^{3+}$$

(2) $${O_{2}}^{∙-}+\;H_{2}O_{2}\to HO^{∙}+\;HO^{-}+\;O_{2}$$

The most common ROS-generation pathway involving the NPs is HO^•^ generation through a photocatalytic process where photon absorption is attended by electron transfer to the conduction band [23,59]. When the light irradiation energy received by TiO_2_ NPs and ZnO NPs is greater than or equal to the bandgap, the electrons in the band are triggered and shifted to the conduction band, leading to hole formation in the valence band (H^+^) [7]. H^+^ interacts with water or OH^−^ and adheres to the surface of the NPs and is then oxidized to the HO^•^ [7]. A similar process occurs when H^+^ interacts with O_2_, where HO^•^ is reduced to O^2ׄ•–^. Ag NPs have been reported to exhibit enhanced antimicrobial activity against *S. aureus* after being radiated with laser energy due to excitation and oscillation of electrons [22]. A study reported a higher ROS level for bacterial cells upon exposure to 0.5 mg/mL TiO_2_ NPs; additionally, a higher still level of ROS was reported when ultraviolet (UV) illumination was used, as evidenced by the greater intensity of red light (indicating dead cells) than green (indicating surviving cells) under confocal laser microscopy (CLSM) [62].

Zhang et al. studied the potential of Au NPs, Ag NPs, silicon (Si) NPs, and nickel (Ni) NPs in aqueous suspensions for ROS generation under UV irradiation at 365 nm [63]. Their results revealed that Ag NPs displayed excellent antimicrobial activity and generated HO^•^ and O_2_ׄ^•–^, whereas Au NPs, Si NPs, and Ni NPs generated ^1^O_2_, which invaded cells and exerted an antimicrobial effect. However, when Ag NPs alone came in contact with bacteria, they would tend to agglomerate and lose their active surface areas, leading to poor antimicrobial activities [64]. To resolve this, combinations of Ag NPs with other materials such as GO to create nanocomposites to mitigate agglomeration have been studied against various bacterial strains, demonstrating promising antimicrobial activities [63–66]. Mahmoudi et al. stated that Ag/GO nanohybrids afforded excellent antimicrobial properties which prevented bifouling on the surfaces of polysulfone membranes [67]. Additionally, the use of UV light instead of visible light has been found to improve the antimicrobial activity of NPs. The underlying rationale is that UV light can yield sufficient energy for the interband transition to excite the ground-state electrons located at the 4d band to the energy level where O_2_ can seize them and lead to ROS formation, while visible light cannot attain this [68]. A study confirmed that Ag NPs under visible light could not generate ROS though it could promote massive protein aggregation [68]. Furthermore, the diffusion coefficient of ROS is similar to that of O_2_ at approximately 10^5^ cm^2^/s, conferring an advantage to ROS [23]. Considering the thickness of the cell wall and membrane of *E. coli*, the diffusion time will be approximately 10^7^s and the average lifetime of ROS is between 10^5^ and 10^6^s; hence, there will be a sufficient amount of time for ROS to diffuse into bacterial cells [23].

### 2.4 Inhibition of proteins and enzymes

Interactions of NPs with vital proteins cause the latter to lose their important functions and lead to enzyme inhibition. As one of the essential structural, functional, and regulatory components in living cells, proteins comprise hundreds or thousands of amino acids in a long chain, linked by peptide bonds [69]. The manifold functions of proteins include providing mechanical support and immune protection, transporting ligands, transmitting nerve impulses, regulating cell growth, and performing crucial biochemical functions (e.g. respiratory enzymes) [68,69]. Secondary forces composed of Van der Waal’s forces, hydrophobic interactions, hydrogen bonding, and electrostatic forces play a crucial role in stabilizing and maintaining the structure of individual protein molecules [70]. α-helices and β-sheets in secondary protein structures are secured by hydrophobic interactions and hydrogen bonding, while the tertiary structures are maintained by all those said forces [70]. Mechanistically, NPs cause the proteins adsorbed onto them to undergo structural changes [71]. The formation of new secondary bonds and the disruption of the original bonds occur simultaneously during protein adsorption to Au NPs and such changes are said to be irreversible [71]. Structural changes in proteins due to interactions between proteins and peptides binding with NPs can result in chemical denaturation and fibril formation due to thermodynamic instability, subsequently leading to the proteins losing their functions [68,69,72]. The possibility of NPs to interact with proteins is reasonably high as the concentration of proteins in biological fluids can be as high as 35% by volume [73,74].

On the other hand, enzymes are a class of proteins that catalyze diverse biochemical reactions by elevating the rate of reaction in the cells and are crucial in controlling biological homeostasis in living organisms [23]. Blocking enzyme activity is known as enzyme inhibition and is either reversible or irreversible. Enzymes are known to be vital virulence factors during bacterial infections. For instance, bacterial urease produced by *E. coli, K. pneumonia, P. aeruginosa, Enterobacter* spp., *Proteus mirabilis* (*P. mirabilis*) and *Providencia stuartii* (*P. stuartii*), are a virulence factor during urinary tract infections [75]. Hence, the inhibition of these enzymes is an important strategy to tackle infections, as has been well-documented in the literature. In one study, Ag NPs or Au NPs capped with ciprofloxacin, an antibiotic, demonstrated substantial improvement in their inhibition of bacterial urease [76]. In another, ZnO NPs of a small particle size showed strong antimicrobial activity against MRSA and could potentially inhibit the activity of β-galactosidase (GAL) [77]. Raffi et al. found that the treatment of *E. coli* with Ag NPs inhibited respiratory enzymes, resulting in overproduction of ROS and disrupting bacterial DNA replication, and thus suggesting that NPs had affected DNA polymerase [78]. Au NPs have been determined to interfere with enzyme functions, given their reactivity: their interference with thiol groups present in vital enzymes led to excessive production of ROS and caused cell death [79]. Additionally, Au NPs and Ag NPs have been found to tend to target phosphorus- and sulfur-containing soft bases present in DNA molecules [23]. These interactions between NPs and targeted soft bases both hinder bacteria growth and interfere with the integrity of bacterial cells. Recent studies have collectively suggested that NPs are capable of directly or indirectly inhibiting enzymatic activity and exerting antimicrobial effects.

### 2.5 Oxidative DNA and RNA damage

**Figure 4.**
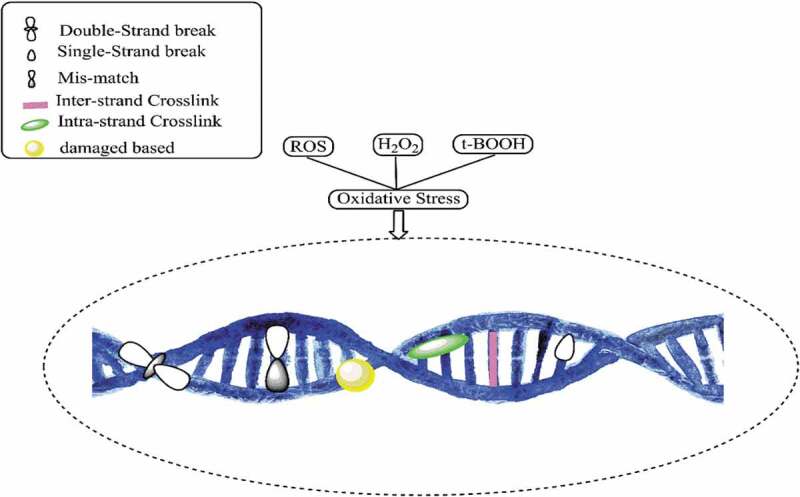
DNA damage due to oxidative stress generated by reactive oxygen species (ROS)

Interactions between DNA and bacterial cells are related to ROS: an overproduction of ROS is generated when exposure to NPs causes oxidative DNA damage [7]. DNA is the fundamental molecule of a living organism. The most crucial functions of DNA include long-term storage of information and transmittance of genetic information for the development of a living organism. The fundamental structure of DNA includes a double-stranded phosphodiester backbone containing genetic codes with four nitrogen bases: adenine (A), thymine (T), guanine (G), and cytosine (C). The breakage of DNA strands is known to be the standard biomarker indicating DNA damage, which can occur naturally or can be induced by other factors. Naturally occurring DNA damage that occurs naturally is considered as common and is not as severe as induced DNA damage because there are cellular adaptive mechanisms to mend the damage and combats any further impacts that may threaten the cells. Conversely, induced DNA damage is caused by various agents, e.g. radiations and chemicals which could be fatal to the cells [23]. DNA damage caused by NPs is considered to be induced by DNA damage and is accomplished through inhibition of DNA replication [80]. Research has shown that upon their diffusion through cell membranes in mammalian cells, Pt NPs promote DNA damages by inhibiting DNA replication and then cause apoptosis [8]. NPs may alter the capacity of the cells to repair induced DNA damage due to the overproduction of ROS. DNA is particularly sensitive to oxidative damage since the production of HO^•^ from Fenton reactions may attack the nucleobases or the sugar-phosphate and lead to saccharide fragmentation aimed at the strand break [81]. ROS also oxidizes the deoxycytidine triphosphate (dCTP) and deoxyguanosine triphosphate (dGTP) causing DNA damages to the 2-deoxyribose moiety of the DNA and DNA bases, resulting in the formation of oxidized DNA bases, single-strand break (SSB), and also the most frequent spontaneous lesions in DNA called abasic (PA) sites [82,83]. NPs can also initiate a double-strand break (DSB) by modulating replication forks, which further leads to apoptosis [84]. Figure 4 depicts DNA damage caused by oxidative stress due to the overproduction of ROS. Compared to oxidized DNA and SSBs that can be repaired by healthy cells, DSB tends to be more severe as it can result in chromosomal aberrations if left unrepaired [82]. Chromosomal aberrations are defined as abnormalities in the number or structure of chromosomes and are often responsible for genetic disorders. It has been reported that, when Ag NPs intercalate with DNA bases, they interrupt the hydrogen bonds between the parallel strands causing DNA to denature, and at the same time can restrain antibiotic-resistant bacterial strains [85].

DNA oxidative processes often target guanine due to its low redox potential, with 8-oxo-7,8-dihydro-2ʹ-deoxyguanosine (8-oxo-dG) as the major degradation product [86]. Eight-oxo is the most abundant DNA lesion and has been established as a biomarker of oxidative DNA damages to quantify oxidative stress damage [87]. Other than that, 8-oxo-dG can mispair with adenine and leads to a transversion where a C:G base pair is misplaced with A:T base pair [88]. 8-oxo-dG also causes oxidative stress in RNA by inducing protein aggregation, and protein mistranslation, thus leading to cell damage [89]. It has been documented that protein aggregation and mistranslation could not only affect bacterial growth rate and stress resistance but also induce cell death [90]. The induction of 8-oxo-dG in cells exposed to NPs has been well-researched. A study demonstrated that 8-oxo-dG levels in wild-type mice did not change after being exposed to Ag NPs for 3 days, though it soared after being exposed for 7 days; elevation trend continued with the level returning to the baseline after being exposed for 14 days [88]. The rising levels of 8-oxo-dG in Ag NP-treated wild-type mice could partly be attributed to the down-regulation of DNA glycosylases responsible for repairing 8-oxo-dG [91]. Gurunathan and colleagues reported that 8-oxo-dG levels in the DNA pool increased three times upon the addition of Ag NPs compared to those of untreated cells and increased drastically 12 times in RNA pool [92]. The greater extent of the increase in the RNA pool suggested more severe damage.

## 3. Antiviral mechanism of nanoparticles

**Figure 5.**
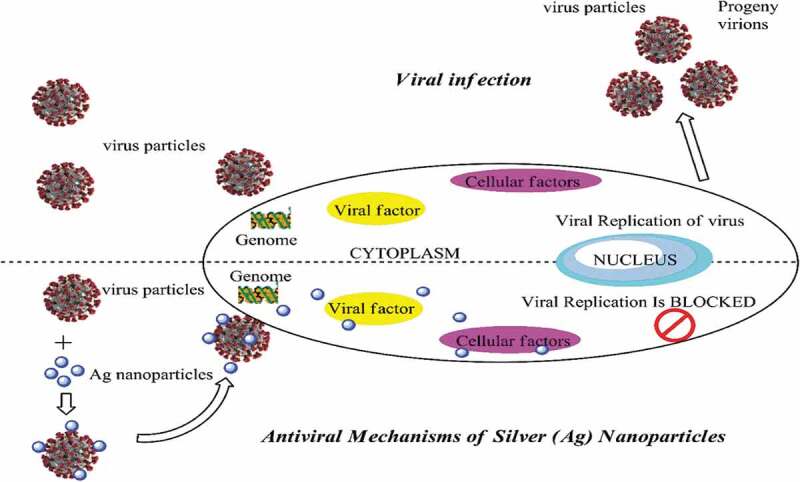
Antiviral activity of Ag nanoparticles

Apart from their excellent antimicrobial activities, NPs exert antiviral effects that underlie their potential as antiviral agents in therapeutics. Viruses are highly pathogenic since they can generate more virulent virus strains by altering the genomes of non-virulent strains. Statistics from the World Health Organization (WHO) revealed that approximately 3 to 5 million cases and close to 650, 000 deaths occur worldwide yearly due to seasonal influenza [92]. Examples of well-known viruses that have emerged in the past few years were severe acute respiratory syndrome-related (SAR) coronavirus, Hendravirus, Hantavirus, Chikungunya virus, and, most recently, Coronavirus (COVID-19). Most viruses possess a unique genetic adaptability through which they can escape antiviral inhibition [93]. Countless attempts to have been made in previous years to develop drugs and vaccines to combat viral and pathogenic attacks. Currently, researchers have focussed on developing potential nano-materials given their remarkable physicochemical properties and high surface-area-to-volume ratio that give them antiviral characteristics. The size of NPs itself plays an important role in their antiviral properties since the direct contact between NPs and viral genomes appears to crucially dictate the antiviral activity of NPs. Brandelli et al. found that NPs of a smaller size would favor interactions between NPs and the virus, thus facilitating viral inhibition [94]. Smaller NPs have been established to easily penetrate the virus-host cell and then enter the genome of the virus and induce viral replication by blocking cellular factors and viral factors mechanism in the genome [94]. Figure 5 illustrates the antiviral effects of Ag NPs.

Microscopic observations have revealed that NPs cause local transformations on viral surfaces, especially the agglutination of glycoproteins upon absorption by the virus. Such a situation hinders viral penetration into the cell [95]. Another antiviral mechanism possessed by NPs is due to the inhibition of cellular factors that are essential for viral progenies [96]. Elbeshesy et al. demonstrated that Ag NPs exerted an antiviral effect on the Bean yellow mosaic virus by binding to the CD4 disulfide bond regions within the viral envelope glycoprotein of the yellow mosaic [97]. Furthermore, interactions between Ag NPs and viral nucleic acids contribute to the antiviral activity as well. Compared to Ag ions themselves, Ag NPs exhibited greater antiviral activity and smaller-sized NPs yielded the same effect, but larger ones did not [98]. A study found that 50 μg/mL Ag NPs demonstrated the optimal antiviral activity with a 79% reduction in respiratory syncytial virus (RSV) replication in A549 cells (human alveolar epithelial cells) and 78% in human epithelial type 2 (HEp-2) cells (originated from a human laryngeal carcinoma) [93]. Moreover, results revealed that the RSV replication in the lung tissues of infected mice was reduced by 55% after being treated by 4 mg/kg Ag NPs [96].

Other than Ag NPs, Au NPs have been used as a carrier of antiviral drugs to prevent viral binding to receptors on cell surfaces. In one study, Au NPs have been shown to prevent the entry and attachment of human immunodeficiency virus (HIV) onto leukocytes and to inhibit early stages of HIV replication [89,99]. In another, polysulphate Au colloids have been demonstrated to inhibit vesicular stomatitis virus (VSV) bonding and infection, while the unmodified Au NPs were unable to interact with the virions [99]. It is noteworthy that combinations of NPs and other potential materials also display antiviral activity. For instance, the antiviral activity associated with mercaptoethane sulfonate for Ag NPs and Au NPs have been tested against type 1 wild herpes simplex virus (HSV): they blocked the attachment of the virus to host cells based on their ability to mimic heparan sulphate from the cell-surface receptor through to the binding of HSV-1 glycoprotein C to heparan sulphate [94]. Moreover, Ag/GO nanohybrids also showed excellent antiviral activity after being incubated with feline coronavirus solution that has been serially diluted. The two conclusions were: 0.1 mg/mL of Ag/GO nanohybrid demonstrated the potential to inhibit the infection of feline coronavirus by 24.8% and a combination of Ag NPs and GO exhibited higher efficacy than GO alone [100]. The underlying reason is that GO could inhibit only enveloped viruses, whereas Ag/GO nanohybrids could prevent infections from both enveloped and non-enveloped viruses [100]. These findings collectively suggest that, besides their potent antimicrobial properties, NPs exhibit substantial potential in their antiviral properties to combat harmful viruses.

## 4. Noble metals nanoclusters

**Figure 6.**
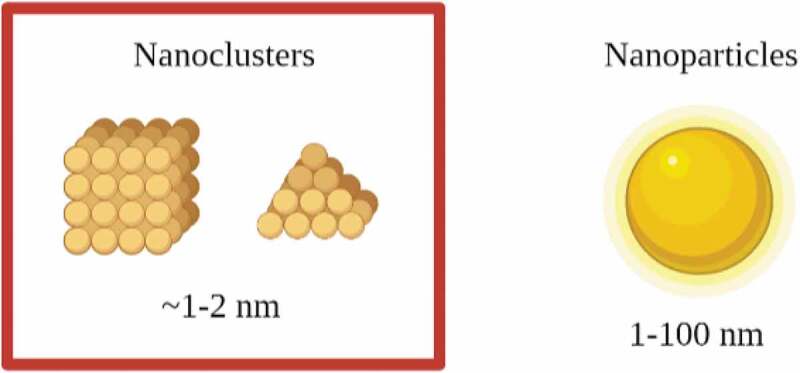
Size comparison between nanoclusters and nanoparticles

Recent advancements have also been focussed on the application of noble metal nanoclusters (NCs) including Ag NCs and Au NCs as antimicrobial agents to combat bacterial infections. Metal NCs are a novel class of luminous NPs that are produced by reducing the size of metal NPs to a size that is equivalent to the Fermi wavelength of an electron with a core size smaller than 2 nm [101]. The size comparison between NCs and NPs is shown in Figure 6. These metal NCs exhibit distinct molecule-like physicochemical properties, such as well-defined molecular structure, quantized charge, molecular chirality, molecular magnetism, highest occupied molecular orbital (HOMO)-lowest unoccupied molecular orbital (LUMO) transitions, and powerful luminescence due to their ultrasmall size [102]. Owing to their unique properties, metal NCs have been widely utilized in biomedical applications, for example, biocatalysis, biosensing, cancer therapy, drug delivery, etc. [103]. The difference between NPs and NCs is that the inherent cytotoxicity of NPs for human cells limits their biomedical application [104]. The cytotoxicity of NCs, on the other hand, can be controlled by cautious selection of nanocore size and shape, as well as the chemical ligands attached to the surface of NCs [105].

Due to NCs relatively small size, they can easily penetrate the cell through the cell wall and across the cell membrane to reach the targets in the bacterial cell. Previous research reported that Ag NCs released Ag^+^ after contact with an aqueous solution, similar to Ag NPs [106]. Hence, it can be concluded that the antimicrobial of Ag NCs may be described by Ag^+^ action, which involves Ag^+^ interacting with the functional group’s enzymes and proteins that will further induce cell process inhibition [106]. In summary, Ag NCs may process the same mode of antimicrobial action as Ag NPs by penetrating bacterial cells, causing overproduction of ROS, damaging essential cellular components in the cell, and ultimately causing bacterial cell death. Moreover, it is also reported that the overproduction of ROS is one of the key factors in determining the antibacterial activity of Au NCs [103]. Zheng et al. mentioned that more negatively charged Au NCs might produce extra ROS that will result in bacterial metabolism disruption and cell death, however, less negatively charged NCs that have amine groups attached on the NCs surface might restrict the production of ROS, hence reduce the bacterial killing ability [107].

### 4.1 Noble metals NCs with different surface capped and its antimicrobial action

**Figure 7.**
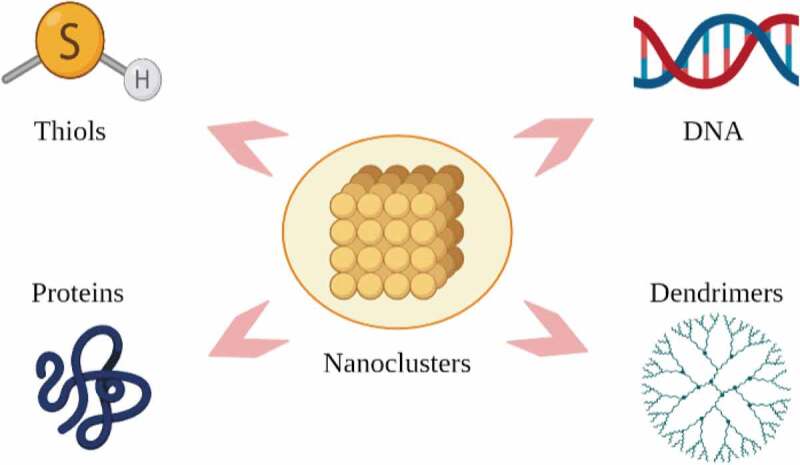
Stabilizers for nanoclusters

The surface properties of NCs are protected by various surface capped such as ligands, proteins, DNA, and dendrimers as shown in Figure 7. The various surface capped may influence the ability of their ROS production, which can directly affect their antimicrobial efficacy [107]. A study revealed that Au NCs that are capped by p-mercaptobenzoic acid (MBA) which are more negatively charged with zeta potential value (ζ = – 36 mV) would greatly induce ROS production in both S. aureus (~2.2-fold) and E. coli (~2-fold). Meanwhile, Au NCs capped by glutathione (GSH) which are less negatively charged with zeta potential value (ζ = – 21 mV) generated a smaller amount of ROS [103]. Facing a similar problem as NPs, NCs also tend to agglomerate into large lumps. Ligands such as thiolates, phosphines, and alkynyls are commonly used to cap the surface of NCs. These ligands act as a cap to hinder agglomeration from taking place and to facilitate the isolation of target Ag and Au NCs. Moreover, these ligands play an important part in the formation processes of NCs, regulating their shapes, sizes, and their properties [102].

Ligands which are small molecules containing thiol, hydroxyl, and amine groups have been utilized in the synthesis of NCs in a method known as the “one-step synthesis method’ where the procedure is separated into two stages. The reducing activity of thiol converts Au^3+^ to Au^+^ in the first stage, forming Au^+^: SR complex, where S is sulphur and R is possibly an alkyl. A powerful reduction agent such as sodium borohydride (NaBH_4_) is used in the second stage to reduce gold to Au. The Au: SR complex is able to form a specific aggregation, preventing the generation of larger particles and at the end of such process, Au is obtained [108]. Ligands as a specific capping agent for NCs are said to form a promising antimicrobial agent due to facile modification, low cost, and high biocompatibility. The antimicrobial action of functionalized Au NCs protected by 6-mercaptohexanoic acid (MHA-Au NCs) was tested on *S. aureus* where the bacteria-killing efficiency was approximate ~95%. Meanwhile, the bacteria-killing efficiency for *E. Coli* was ~96%. The antimicrobial efficacy of MHA-Au NCs is attributed to their relatively small size, which allows for better contact with bacteria compared to Au NPs. The interaction of MHA-Au NCs with bacteria causes a metabolic imbalance, resulting in a higher ROS production, which eventually leads to cell death [109].

Proteins are known as biomacromolecule ligands and are said to have specific binding sites for metal ions which contribute to specialized templates for the synthesis of NCs. Ying and co-workers previously synthesized Au NCs with the use of bovine serum albumin (BSA), where Au^3+^ is collected by BSA. Following tyrosine and cysteine residues in the structure of the protein molecules reduce those ions to atoms. BSA also gives advantages to Au NCs as it helps Au NCs to improve their stability by providing structural support. Moreover, temperature plays an important role in the synthesized process of BSA-capped Au NCs and the temperature similar to the human body is proven to be the best as temperature lower or higher than that will affect Au NCs nucleation where if the temperature is too low, Au NCs will be hard to nucleate. Meanwhile, if the temperature is too high, Au NCs will be unstable and formed large lumps due to agglomeration [108]. BSA-capped Au NCs antimicrobial action is due to BSA being constituted in a hollow cylinder with an open channel which gives access to ROS to attack the metal core of BSA-capped Au NCs and induce more ROS that leads to bacterial death. Moreover, Au NCs have a high affinity towards sulphur and form a covalent bond with sulphur. The formation of covalent bonds results in protein dysfunction in bacterial cells. This antimicrobial action of BSA-capped Au NCs is proven when the absorbance value (OD600) of treated bacterial with BSA-capped Au NCs were half lower (between 0.07 and 0.22) compared to the absorbance value of untreated bacteria (between 0.52 and 0.75) [110].

Besides that, studies on developing DNA-capped metal ions/complexes have also gained so much attention on research due to the DNA base sequence’s tunability which makes it unchallenging in synthesizing NCs with different particle sizes and degrees of dispersion [108]. Compared to Au NCs, there are more studies on DNA-capped Ag NCs (DNA-Ag NCs) as Ag NCs favor to be functionalized with thymine- or cytosine-rich single-stranded DNA owing to the strong interaction between Ag and the N3 of thymine or cytosine [111]. Previous studies revealed that Ag NCs in an aqueous solution can be formed at room temperature by using a 12-base scaffold of 5ʹ-AGGTCGCCGCC-3ʹ which was used as a template for the assembly of Ag^+^ where it was further reduced by NaBH_4_ [102]. Ag NCs have strong antimicrobial action; however, DNA-Ag NCs with an aptamer have more effective fast-acting antimicrobial action as it can kill 50% of *the P. aeruginosa* population within 10 minutes post-treatment by disrupting the bacterial cell membrane to increase bacterial cell permeability that will reduce the membrane potential [112]. Furthermore, the combination of modified vancomycin-stabilized fluorescent Au NCs (Au NCs@Van) and aptamer-modified Au NPs increases the sensing of S. aureus using a dual-recognition motif-based fluorescence resonance energy transfer (FRET) platform, which has a lot of potential in terms of diagnosing infectious diseases [113].

Metal NCs can also be encapsulated and functionalized using dendrimers as dendrimers containing a variety of functional groups, making them potential templates for the synthesis of metal NCs with strong biocompatibility and high water solubility [111]. The functional groups of dendrimers also contribute a rich active site for the reaction of the synthesis of metal NCs to proceed, meanwhile, the internal pore cavity isolates the metal ions from the solution, preventing NCs agglomeration [108]. Moreover, dendrimer-capped NCs have steric protection provided by the dendrimer shell which can improve the stability of metal NCs, allowing them to be further functionalized via chemical conjugation and electrostatic interaction for transporting therapeutic agents purposes [111]. Poly(amidoamine) (PAMAM) dendrimers have been utilized to functionalize Au and Ag NCs where the synthesized NCs were extremely stable in solution and managed to produce high quantum yield. Au8 synthesized using PAMAM has strong blue fluorescence, with a quantum yield of 41% in an aqueous solution, which is a hundredfold increase [108].

## 5. Nanotechnology-based antimicrobial applications

The manifold remarkable properties of various NPs have contributed to an array of their nanotechnological applications in diverse fields, particularly in the medical and pharmaceutical realms. However, NPs have also been used in a wide range in the food sector, water treatment, cosmetics ingredients, antimicrobial textiles, and many more [79,114,115]. The application of nanotechnology-based antimicrobial agents in the medical and pharmaceutical fields has been explored due to the excellent antimicrobial activities of NPs [7].

**Figure 8.**
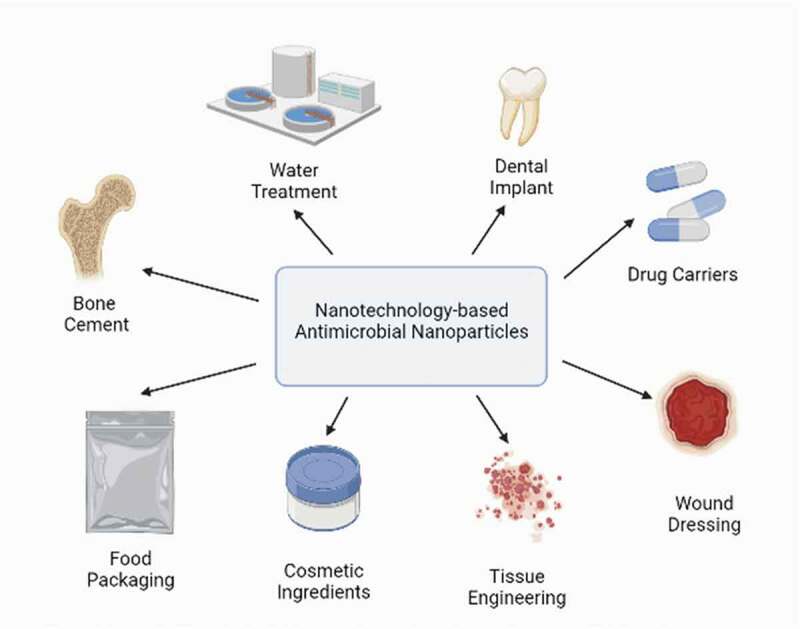
Nanotechnology-based practical applications

Among the applications of NPs (Figure 8), the use of NPs in wound dressing has received tremendous scholarly attention. Epidermal injuries, if untreated, could lead to wound infections due to the penetration of microorganisms from the surroundings as the skin not only is one of the most important protective barriers from pathogens and harmful substances from outside the body but also maintains the dynamic equilibrium of water and electrolytes in the body [116]. Recent studies have shown that antimicrobial wound dressings have been viewed as an alternative approach in reducing bacterial colonization and infection in wound healing [117]. The favorably small particle size of NPs increases their surface-area-to-volume ratios, resulting in superior physicochemical properties ideal for their integration into wound dressings [118].

Ag NPs can regulate the release of anti-inflammatory cytokines to encourage fast wound healing without deepening scars, but they also boost wound contractility by inducing different myofibroblasts from normal fibroblasts, thus hastening to heal [119,120]. However, there is a limitation since the higher concentration of Ag NPs reduces keratinocyte viability, metabolism, and migratory potential of these cells; it may also activate caspase 3/7 and trigger DNA damage due to longer exposure [121]. Combinations of low-dose Ag NPs and other antimicrobial agents have been determined to minimize the side effects associated with using Ag NPs alone. In one study, the combination of Ag NPs and tetracycline synergistically reduced the bacterial load in murine superficial and deep tissue layers [122]. In another, the combination of Au NPs and basic fibroblast growth factor (bFGF) in various concentrations of Vaseline mixtures promoted fast wound closure caused by the stimulation of angiogenesis and fibroblast proliferation without causing cell toxicity after undergoing treatment for 14 days [123]. These findings encourage the combination of NPs with other antimicrobial agents to more effectively treat infected wounds.

Besides wound healing, NPs have found potential utility in tissue engineering, where a chief challenge in tissue replacement due to trauma or diseases is the inability of materials to imitate the natural properties of tissues [124]. The use of scaffolds for the regeneration of tissues has been known to be the major research field in tissue engineering [125]. One of the desirable characteristics of scaffolds in tissue engineering is a high surface-area-to-volume ratio which facilitates cell adhesion, migration, proliferation, migration, and differentiation. Accordingly, nanotechnology through customized engineering of NPs has been cited to offer practical utility due to their high surface area [126]. Evidence of the application of NPs in tissue engineering has copiously been reported. In one study, the high specific area of Ag NPs has been found to attach to bacteria easily, hinder their development, and hasten the wound healing process [7]. In another, NPs have been determined to provide controlled release of bioactive agents and improve the mechanical strength of scaffolds [127,128]. Moreover, it is noteworthy that the incorporation of NPs enhances the properties of scaffolds. In terms of mechanical properties, the incorporation of Au NPs has been shown to not only improve the adhesive properties of scaffolds, but also stimulate cell proliferation and differentiation [125]. Additionally, their incorporation into nanofibrous scaffolds can alter the cell-material interfaces and improve the affinity and stability of scaffolds [129]. In terms of antimicrobial properties, NPs incorporated in scaffolds comprising different materials such as gelatin, poly(vinyl alcohol) (PVA) and chitosan exhibited potent activities against bacteria [130]. Lastly, in terms of biochemical properties, one study has reported that combinations of bifunctional scaffolds with antibacterial composites such as Ag/GO nanohybrids promoted osteogenic actions for the repair of large-bone defects while preventing or treating infections [131]. Another study has revealed that antimicrobial scaffolds comprising chitin composites incorporated with Ag NPs altered coagulation pathways through denaturing anticoagulant proteins, thus improving blood clotting efficiency [130].

The application of NPs in bone cement has been shown to exert potent bactericidal effects on antibacterial-resistant bacteria [7]. Extensively used for implant fixation in orthopedic and trauma surgery, bone cement is a biomaterial that serves to fill in the gap between bones and implants. Two types of bone cement are available: calcium phosphate and (the more commonly used) polymethylmethacrylate (PMMA) [132]. The clinical significance is reflected by the estimated 35,000 cases of post-operative infections, thus underlining the severity of such complications related to orthopedic procedures [133]. Numerous encouraging findings have been reported. PMMA-based bone cement incorporated with Au NPs has been proven to minimize the formation of biofilms of *S. aureus,* whereas those with Ag NPs likewise minimized biofilms based on the Kirby–Bauer method by preventing bacterial colonization [134,135]. Furthermore, the number of arthroplasty-related infections due to bacteria including *S. aureus, S. epidermidis, A. baumanii*, and MRSA has been shown revealed to reduce drastically with Ag NPs (at concentrations as low as 0.05%) [136]. The long-term antibacterial effect was tested on *Enterococcus faecalis* (*E. faecalis), Enterobacter cloacae* (*E. cloacae), P. aeruginosa, S. aureus*, and *S. epidermidis* for bacterial adhesion for 14 days. The results displayed a reduction in bacteria biofilm formation on the bone cement containing antibiotics but bone cement containing NPs showed better effects where the reduced rate is much higher [137]. In addition, NPs are known for their application as coatings for medical devices due to their unique properties.

The use of urinary catheters to drain a patient’s bladder can cause the spreading of catheter-associated tract infections (CAUTIs). Pathogens use the catheters as a podium for their colonization and formation of biofilms, resulting in bacteriuria and elevated chances of secondary bloodstream infections [138]. Biofilms act as protectors for microorganisms by defending them from antimicrobial agents and antibiotics. *E. coli* has been found to be the main infective microorganism for CAUTIs and it is estimated the rate of CAUTI is about 5% per day [139]. Statistics have revealed that CAUTIs affecting approximately 15% to 25% of hospitalized patients [140]. For urinary catheters coated with Ag NPs, the dissolution of such NPs resulted in Ag^+^ release which in turn induced the formation of ROS; such catheters displayed outstanding activities against both Gram-positive and Gram-negative bacteria by inhibiting both the formation of biofilms and microbial growth [141]. Compared to Ag-coated catheters, Ag-polytetrafluoroethylene (Ag-PTFE) coating for catheters exhibited superior antimicrobial activities: it reduced bacterial adhesion by up to 60.3% and reduced the formation of biofilms by up to 97.4% [142]. However, despite the excellent antimicrobial activities, the leaching of NPs from the catheters remains a concern [143]. Besides their application to catheters, NPs have been used as coatings for various medical devices to reduce healthcare-associated infections (HAIs). Cotton coated with CuO NPs and polymeric substrates present remarkable antibacterial properties on *E. coli* and *S. aureus* even after undergoing 30 washing cycles [144]. Photo-enhanced antibacterial effects of 0.5 mg/mL TiO_2_ NPs have been found to induce changes in bacterial morphology: the bacteria were observed in red fluorescence channels rather than in green under a confocal image, indicating a higher rate of membrane polarisation and bacterial death [62]. This finding has suggested the antibacterial utility of TiO_2_ NPs in hospital garments as it can manage bacterial infections. Siberian ginseng associated with Ag NPs has been employed in disinfecting surgical instruments, therefore avoiding cross-contaminations and enhancing safety in hospitals [145].

In dentistry, the use of nanotechnology-based antimicrobial NPs has been on the rise. NPs have been used to combat dental caries by integrating antimicrobial NPs into composite resins to minimize biofilms [146]. Such achievements in resisting dental caries have been proven by Ag NPs and ZnO NPs embedded into a composite resin, resulting in the growth inhibition of *Streptococcus mutans* (*S. mutans*) and *Lactobacillus* [147]. In order to minimize the possibility of implant failures, dental implants have been modified with NPs such as TiO_2_ NPs which provided mechanical strength and improved antimicrobial properties of tooth-filling materials [135]. In one study, the incorporation of 2.5% ZnO NPS into PMMA has been found to exert an antifungal effect; in another, colloidal dispersions and irrigating solutions used for disinfecting root canal consisting of NPs-H_2_O_2_ assemblies have demonstrated its bacterial activities on *E. faecalis* compared to conventional dental antibacterial irrigants [135,148]. Due to reduced pH and bacterial proliferation in root canal treatment, orthodontic treatment almost invariably results in dental plaque chalk formation. Brackets coated with ZnO and with CuO NPs can significantly hinder bacterial plaque development, though such coating may affect the appearance of the brackets [7].

NPs have also been employed in oral prevention methods as antimicrobial agents. TiO_2_ NPs have been demonstrated as an active antimicrobial agent in mouthwashes [147]. In their study on *S. mutans*, Ahmed et al. found that the in vitro zones of inhibition with a mean diameter of 20.14 ± 0.96 mm were observed for the Ag NP-incorporated toothpaste, compared to the no zones being observed for the toothpaste without NPs [149]. Toothpaste with Ag NPs at low concentrations also has the potential to completely inhibit the growth of *E. faecalis* and *Bacillus cereus* (*B. cereus*) [150]. In the food industry, NPs have found practical utility in packaging and storage due to their ability to control microbial contamination in foods [79]. Microbial contamination is the chief concern as it causes huge wastage of foods, compromises the quality, shortens the shelf life, and leads to public health problems [151]. The most common foodborne pathogens are *Campylobacter jejuni* (*C. jejuni), Salmonella Enteritidis* (*S. Enteriditis*), and *E. coli* [152].

Nowadays, NPs with better abilities have been introduced as they can withstand extreme processing conditions such as exposure to intense temperature, improve food quality, and prevent microbial adhesion on foods [153]. Among all NPs, Ag NPs have been the most studied NPs due to their excellent stability and antimicrobial activity [154]. Shankar and Rahim produced antimicrobial packaging materials by integrating Ag NPs with poly(butylene adipate-co-terephthalate) (PBAT), which improved vapor permeability and exerted a bactericidal effect on *L. monocytogenes* and *E. coli* [155]. Wrappings in low-density polyethylene (LDPE) films with Ag NPs have been found to lengthen the sustainability of chicken fillets [154]. Moreover, the exposure of the films to UV radiation for 20 min exhibited a bactericidal effect on *Pseudomonas fluorescens* (*P. fluorescens*) and *S. aureus* [156]. Other than Ag NPs, the incorporation of other NPs such as ZnO NPs and MgO NPs have been researched. Petchwattana et al. studied the ability of poly(butylene succinate) (PBS)/ZnO composite films for food packaging with different concentrations of ZnO NPs: they found that PBS/ZnO composite films with 6 wt% ZnO NPs hindered the growth of *E. coli* and *S. aureus* with the zones of inhibition measuring 1.31 and 1.25 cm, respectively [157]. It was also confirmed that 2 mg/mL MgO NPs is required by *C. jejuni*; meanwhile, 8 mg/mL MgO NPs concentration is needed for *E. coli* and *S. Enteriditis* to entirely inactivate 10^8−9^ CFU/mL due to induction of oxidative stress from ROS released by MgO NPs [152]. In food processing, TiO_2_ NPs and silicon dioxide (SiO_2_) NPs are accepted as food additives in bulk quantities known as E171 and E551, respectively [158].

In wastewater treatment, nanotechnology-based antimicrobial NPs can offer practical value in the utilization of treated wastewater or safer disposal. Enhanced water quality has been reported through procedures such as chlorination, UV treatment, and ozonation, but they have limitations. Chlorination is not effective against some highly resistant waterborne pathogens [159]. The UV treatment provides no protection against the re-infection process; ozone tends to form deleterious bromate upon reaction with bromide ions in water [160]. The integration of NPs in wastewater treatment is mainly because of their small sizes: the resultant large surface areas offer them strong reactivity and adsorption capacities [161].

Despite the potential in wastewater treatment, the direct use of NPs in wastewater can cause them to agglomerate [162,163]. Therefore, NPs need to be encapsulated onto supporting materials to reduce agglomeration since agglomeration will compromise both their stability and antimicrobial properties [161]. One study found that Ag NP-embedded Poly(2-hydroxyethyl methacrylate-glycidyl methacrylate), (Poly(HEMA-GMA) for short) cryogels reduced the number of *E. coli* in samples after water filtration and the average size of the Ag NPs used was smaller than 100 nm [164]. The combination of Ag and GO is also proved to reduce the agglomeration of Ag NPs. GO is a two-dimensional material made up of a single hexagonally arranged layer of sp^2^-bonded carbon atoms with large surface areas and numerous oxygen-containing functional groups such as hydroxyl, epoxy, and carbonyl groups protruding from GO backbones, which make the GO more hydrophilic [165,166]. Thus, it offers excellent dispersion of NPs on the edges of its planes through chemical bonding even at high concentrations. Wierzbicki et al. (2019) identified three peaks associated with agglomeration in the particle size distribution of Ag NPS, but only one peak in that of Ag/GO nanohybrids, thus concluding the anti-agglomeration effect of Ag NPs with GO [167]. Another study determined that Ag NPs at a concentration of 676.9 mg/L and, encapsulated with polyurethane foams exhibited strong antimicrobial activity: the number of *Fecal streptococci* (*F. streptococci*) and *S. aureus* for wastewater treatment dropped to zero after a contact time of 60 minutes and 120 minutes, respectively [168,169]. In addition, Cu NPs coated with polyurethane foams and Cu NPs encapsulated in alginate beads have been developed as antimicrobial filters against *E. coli* for water purification [135].

## 6. Biocompatibility of nanoparticles

Investigations of the biocompatibility of NPs are warranted since their biomedical applications such as drug delivery, biosensing, treatment of wound infections, and dentistry necessitate their direct contact with in vivo cells and tissue. The toxicity of NPs can be assessed via the in vitro and in vivo methods [8]. Herein, several NPs and their effective roles are discussed. The cytotoxicity of mouse fibroblast cells was tested through a direct contact method on Ag-coated catheter and Ag-PTFE-coated catheter: for both catheters, the cell viabilities were higher than 82% but, for Ag-coated catheters, the increasing incubation time caused the cell viability to decrease slightly. Different results were obtained for Ag-PTFE-coated catheters, where the cell viability remained high and stable throughout the incubation, suggesting that the biocompatibility of Ag-PTFE-coated catheters improved when NPs were encapsulated with other materials [134]. Moreover, the exposure of human and mammalian cells to Ag NPs at low concentrations (10 µg/mL) for 24 hours has been found to exert mild oxidative stress on the cells as their viability declined by 30% [78]. Cells treated with Ag NPs with such low concentration recover their growth upon transfer to a fresh medium without Ag NPs.

Xiang et al. proposed a method to boost the antimicrobial action of titanium (Ti) implants through coating with poly(lactic-co-glycolic acid)/ZnO nanorods/Ag NPs and their results revealed that the cytotoxicity of PLGA-ZnO-AgNPs was lower than ZnO and ZnO-Ti against mouse preosteoblasts (MC3T3- E1) cells [166]. In another study, Ti alloy (Ti6Al4V) dental implants were coated with Ag NPs, together with hydroxyapatite (HA) and 3 samples were prepared: Ti6Al4V with Ag NPs, Ag NPs plus HA microparticles (Ag+mHA), or Ag NPs plus HA NPs (Ag+nHA) [157]. The osteoblasts tested retained their normal morphology and cell viability was close to 70% throughout treatment with Ag+nHA which demonstrated that its biocompatibility compared to the other two Ti implant samples, suggesting its clinical suitability [170]. One of the most reliable methods to measure the biocompatibility of NPs is through induced haemolysis [171]. Haemolysis involves the denaturation of cells through physicochemical interactions between cell surfaces and NPs. The increasing permeability of complete cell lysis influences the mechanism of the haemolytic activity in NPs and cell lysis generates free radicals, resulting in a harmless RBS cell count [168]. Lower haemolytic activity was obtained when red blood cells (RBCs) were tested with ZnO NPs at different concentrations of 25, 50, 75, and 100 μL. Among all concentrations tested, 100 μL ZnO NPs exhibit a higher percentage of haemolysis in both green synthesis and chemically synthesis methods [171]. This finding thus suggests the biocompatibility of ZnO NPs utilized in the recent anticancer and antimicrobial activities.

The biocompatibility of Au NPs has been described in numerous previous studies. In their study on acetaminophen-stabilized Au NPs (A-AuNPs), Rajendran et al. found that the viability for Vero cells was observed to be 97.8% for the minimum concentration (50 μg/mL) of A-AuNPs and 86.2% for the maximum (250 μg/mL). Meanwhile, the viability for A549 lung cancer cells was observed to be 98.6% for the minimum concentration and 90.4% for the maximum. Moreover, neither signs of overt toxicity were present, and the cell viability did not drop significantly at the highest concentration of A-AuNPs [172]. Since the cell viability exceeded 85% for both cells, A-AuNPs can be concluded to be biocompatible, previous researchers suggest that NPs with the ability to maintain cell viability higher than 80% are considered safe to be employed in biological applications [172]. Numerous bio-benign nano-systems have been prepared in which Au NPs appeared as the most biocompatible for drug delivery [173]. In view of the combined biocompatible and cytotoxicity effects of NPs, it can be said that most NPs possess both properties. These desirable properties depend on various parameters including the type of cell, incubation period, size, and dose of NPs used. Moreover, modifications of the biocompatibility and cytotoxicity of NPs can be achieved by changes on their surfaces through encapsulating NPs on other potential materials. However, more research is needed to investigate the ability and consequences of these properties of NPs.

## 7. Bottleneck and future prospects of nanoparticles in antimicrobial actions

The exact mechanisms underlying the antimicrobial activity of NPs have not hitherto been conclusively elucidated. While some evidence has suggested that their antimicrobial activities solely depend on the production of ROS or the development of oxidative stress that leads to apoptosis, other evidence has revealed that the antimicrobial effects are unrelated to metabolism regulations such as MgO NPs [1,7]. Moreover, research is still underway on whether the toxicity of NPs originates from ions released from NPs or the NPs themselves [174]. Hence, the antibacterial mechanisms of NPs in detail are worth taking into account in future research.

Other technical complications have persisted. The lack of unified standards for antimicrobial activity of NPs and variances in parameters such as the types of bacterial strains used, action times, and NPs characteristics have both contributed to difficulties in quantitative comparison. Second, the cytotoxicity of NPs towards living organisms such as humans has remained a concern: evidence on their safety in biomedical applications is lacking since the behaviors of NPs in the human body have yet to be explored. Therefore, these two challenges have to be addressed before the use of NPs as practical therapeutic agents. Third, the uncertainty of ecological effects from the use of NPs represents a challenge: studies have reported the adverse impacts secondary to their discharge into the environment, and such discharges have been projected to rise with the rapid growth of their industrial applications. Hence, future endeavors should focus on mitigating such environmental repercussions.

## 8. Conclusion

Nanoparticles have increasingly been investigated in recent years owing to their unique properties, especially in biomedicine and therapeutics. The diversity of their mechanisms against microorganisms has offered numerous benefits in their antimicrobial activities and their biocompatibility, appealing potential for practical applications in nanomedicine. NPs can be employed for coating in hospitals, medical devices, and operation theatres to minimize infections from bacteria [6]. However, the industrial large-scale applications of NPs can have severe biological effects at cellular and subcellular levels. Similarly, excessive discharges of NPs into the environment can lead to repercussions. Therefore, to meet the rising demands, future work needs to focus more on designing applicable and economical methodologies for scaled-up NPs manufacturers.
